# A Compact 3D Omnidirectional Range Sensor of High Resolution for Robust Reconstruction of Environments

**DOI:** 10.3390/s150202283

**Published:** 2015-01-22

**Authors:** Roberto Marani, Vito Renò, Massimiliano Nitti, Tiziana D'Orazio, Ettore Stella

**Affiliations:** Institute of Intelligent Systems for Automation, Italian National Research Council, via Amendola 122/DO, 70126 Bari, Italy; E-Mails: reno@ba.issia.cnr.it (V.R.); nitti@ba.issia.cnr.it (M.N.); dorazio@ba.issia.cnr.it (T.D.O.); stella@ba.issia.cnr.it (E.S.)

**Keywords:** three-dimensional reconstruction, environmental monitoring, laser profilometry, range sensor, catadioptrics

## Abstract

In this paper, an accurate range sensor for the three-dimensional reconstruction of environments is designed and developed. Following the principles of laser profilometry, the device exploits a set of optical transmitters able to project a laser line on the environment. A high-resolution and high-frame-rate camera assisted by a telecentric lens collects the laser light reflected by a parabolic mirror, whose shape is designed *ad hoc* to achieve a maximum measurement error of 10 mm when the target is placed 3 m away from the laser source. Measurements are derived by means of an analytical model, whose parameters are estimated during a preliminary calibration phase. Geometrical parameters, analytical modeling and image processing steps are validated through several experiments, which indicate the capability of the proposed device to recover the shape of a target with high accuracy. Experimental measurements show Gaussian statistics, having standard deviation of 1.74 mm within the measurable range. Results prove that the presented range sensor is a good candidate for environmental inspections and measurements.

## 1. Introduction

The recent technological developments have opened new ways of inspecting the world in every detail. If a picture is worth a thousand words, a three-dimensional (3D) image addresses much more information about a specific target, or, more generally, the environment.

The first need of 3D data comes from the field of robotics, where exhaustive maps of a robot's surroundings are mandatory for its self-localization and to perform collision avoidance [1–4]. At the same time 3D images, also known as range images, have attracted increasing interest by companies in the field of quality control since the detailed and unsupervised inspection of manufactured goods can speed up industrial processes, especially in those fields, *viz.* automotive and aeronautics, where industrial standardization is mandatory [5–7]. Starting from these problem-driven applications, the use of 3D data has been extended to several additional fields [8,9], spanning from medicine [10,11], geology [12–15] and biology [16], to archaeology [17–19] and reverse engineering [20,21].

In the last decade, many range sensors have been developed and later commercialized for the 3D mapping of indoor and outdoor scenes. Among them, the most used exploit stereo imaging, time of flight principles, structured light, and laser triangulation. Table 1 lists some examples of available systems, displaying the main features, such as the acquisition rate, resolution, accuracy and precision.

Stereo imaging [27] takes advantage of more views of the same target to compute its depth. Several sensors have been proposed (e.g., [25,28,29]), but unfortunately the need of point correspondence among the acquired images and the complexity of the mathematical models required to triangulate these points limit their applicability in actual context of measurement. As a consequence, stereo vision is often used for qualitative real-time analysis of dynamic scenes, such as in [30,31].

Time of flight (ToF) range finders compute the target distance in terms of the time elapsed between an issued laser beam and its reflected spot. Many devices (e.g., [32–34]), also known as lidar, generate a single laser beam which is then deflected by moving mirrors in order to scan wide areas. As an example, the AccuRange AR4000 [22] implements a rotating mirror which reflects light on the environment, describing circular profiles. As a drawback, the use of mechanical components limits the applicability of these sensors since the sample rates decrease. Although a single spot can be acquired at a maximum rate of 50 kHz, actually, this value is limited by the rotating mirror to 1000 samples per second, corresponding to few tens of profiles per second. At the same time (ToF) cameras try to overcome this aspect by adding system redundancy, *i.e.*, increasing the number of detectors (see the commercial products in [35,36]). In this case, the emitting lasers shed light over wider areas, whereas the matrix of detectors compute the phase difference between the sent signals and the returned ones. A depth image having the size of the matrix of detectors is thus computed in a single shot. Increasing the number of camera pixel, the corresponding equivalent sample rate can surge of orders of magnitude. On the contrary, the achievable field of view is implicitly bounded, so that multiple acquisitions are necessary to get a full mapping of the surroundings, with problems residing in the registration of the different unknown views. Moreover the cost of such systems is still impressive because of the number of laser sources illuminating the environment. Further commercial sensors, devoted to the home entertainment (Microsoft Kinect v2 [26]), employ diffused modulated light to illuminate the scene, thus downing the overall costs at expanses of the final measurement resolution.

Terrestrial Laser Scanners (TLSs) are devices used for the modelling of complex targets under outdoor conditions, with maximum ranges of hundreds of meters [23]. Such systems are based on the principles of time-of-flight or of phase difference and typically return range data as a function of the angular position of the emitted laser line. Their typical applications fall in the monitoring of extended areas for the detection of landslides and terrestrial deformations, or in the field of 3D reconstruction of cultural heritage sites [37,38]. However, the main drawbacks reside in the huge cost of TLSs, their dimensions and weights and the limited field-of-view (FoV) which makes them suitable mostly for long range measurements, and often not adaptable for several applications which require environmental scans of complex scenes.

Structured light patterns are often used to compute the 3D shapes of objects, since they are deformed in accordance with the profile of the surface under investigation. Light patterns can be made of stripes (as in [39,40]) or points (see [41]), whose distribution in the camera image is preliminary determined with reference to a calibration plane. Each alteration of the target surface with reference to this plane returns a shift of the detected pattern, depending on the change of depth. The main limit of this technique resides in the mere indoor use, where fringes and spots are highly distinguishable. Outdoor application requires the use of coherent light, such as laser beams.

Laser profilometers follow the same principles of structured light, for which a laser line impinging a target is accordingly deformed. Knowing the relative position of laser and camera, triangulation laws can derive the position of the line in an absolute reference system [42]. As for the ToF range camera, the weakness of this technique is related to the bounded field of view of the sensor, which does not permit the full mapping of the sensor surroundings. For this reason mirrors can be added to collect a wider sight of the environment in a single frame. These complex systems belong to the category of sensors baser on catadioptrics [43–45].

The main idea of the proposed setup has been already presented by the authors in [46–48], where an omnidirectional sensor for high-resolution 3D mapping has been proposed. Here a laser profilometer assisted by a parabolic mirror is designed to reconstruct spaces when a mobile robot flows through them. The achievable resolutions (10 mm at 5 m of distance from the laser source) have demonstrated the capability of the previous approach to precisely model both indoor and outdoor scenes, going beyond the mere 3D mapping devoted to robot navigation and obstacle avoidance. Previous results have enabled novel applications, such as the detection of wall cracks or the prevention of geological hazards, as landslides and rockfalls, just to mention a few.

A step forward in the sensor development consists of reducing the size of the whole experimental setup, without altering the final accuracy. In fact, downsizing the setup enables the possibility of using it in further applications, including pipe inspection and monitoring of dangerous and confined spaces. For this reason the prototype has been completely redesigned with state-of-art devices (lasers and camera) able to further increase the acquisition rate. Furthermore, the calibration phase has been lightened by means of a novel numerical approach for the exact computation of the actual parameters involved in the measurements. In this way, precise mechanical alignment of the optical components which constitute the system is no longer required.

The paper is organized as follows: Section 2 describes the working principles of the proposed sensor, showing its geometry, the analytical model that permits the 3D mapping and the evaluation of the design parameters that meet the initial specification. Section 3 first reports the description of the experimental setup and then collects the discussion of experiments, including a detailed explanation of the calibration phase. Conclusion and remarks are given in Section 4.

## 2. Sensor's Working Principles

This Section aims to describe the working principles of the presented setup, showing how the components cooperate to sense the surroundings. Starting with the description of the setup components, this Section flows through the investigation of the mathematical formulations that lead to the design of the sensor prototype.

### 2.1. Geometry Description

The proposed sensor is designed to map environments with high resolutions and high frame rates, exploiting the principles of laser triangulation. Although profilometry is a rather simple way to retrieve the 3D shape of objects, or more generally of any surrounding, its fundamental limit resides in the short available FoV. In the simplest case of a laser generating a line over the target and a receiving camera, which directly looks at the illuminated surface, the FoV is limited by the sensor width times the lens magnification. Since short-focal lenses are not suitable for measuring because of the huge distortions, the magnification is not enough small to increase the FoV to a full representation of the environment. To achieve this result exploiting the advantages of laser profilometry, it is mandatory to increase the component redundancy or combine one or more mirrors with one or more cameras. These systems are referred as catadioptric systems.

In general, catadioptric systems are made of a standard camera, with perspective or orthographic projection models, pointing upward a convex mirror (parabolic, hyperbolic, conic, *etc.*). As a consequence the FoV of the camera is opened to the surrounding regions beyond the limit imposed by the camera lens. On the other hand, such systems introduce deformations of the acquired images. As a consequence, image distortions have to be compensated to produce effective measurements, taking advantage of the knowledge of the mirror equation.

**Figure 1.**
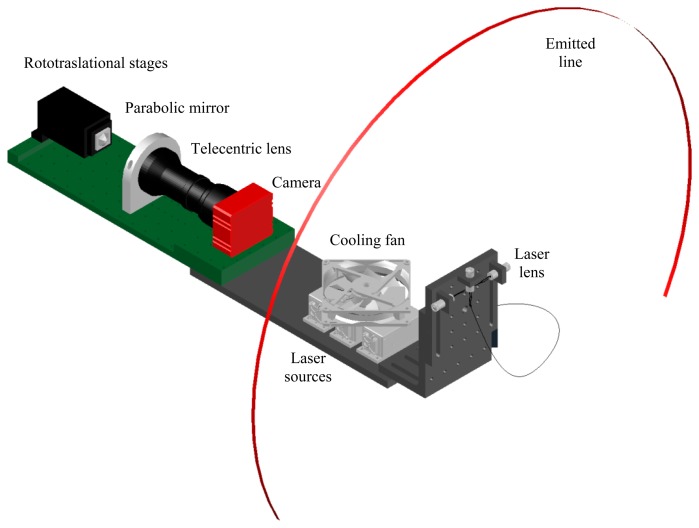
Sketch of the presented laser profilometer. Note the parabolic mirror is mounted onto micrometric rototranslational stages, whereas the camera is fastened on the metallic stand. Lasers are placed across from the parabolic mirror, behind the camera.

Following the approach described in [48], the proposed sensor falls in the category of the catadioptric laser profilometers, since it is made of a laser source, a parabolic mirror and an optical receiver. With reference to Figure 1, three laser sources are used to emit light, forming a plane with an overall fan angle of 270° (90° each laser). When the light strikes a target of the surrounding environment, a complete line is displayed on the surfaces. Each point of the line describes a measurement sample where the scene will be mapped in the global reference system. The parabolic mirror deflects light on the camera plane, throughout the lens. Since a parabolic mirror reflects light always following directions parallel to its axis of symmetry, a telecentric lens is the best candidate for the image formation. The resulting image has information about the position only of the illuminated targets. It is worth noticing that the sensor must be aided by an encoded movement to perform a complete scan of the whole environment. For this reason a mobile vehicle is used to carry the sensor through the scene, sense its spatial pose *via* standard odometry and send this information to the data collector.

Once the fundamental active devices are chosen and arranged in the setup, it is mandatory to derive the triangulation laws that govern the process of image formation on the camera. This aspect will be topic of the next section.

### 2.2. Triangulation Equations

**Figure 2.**
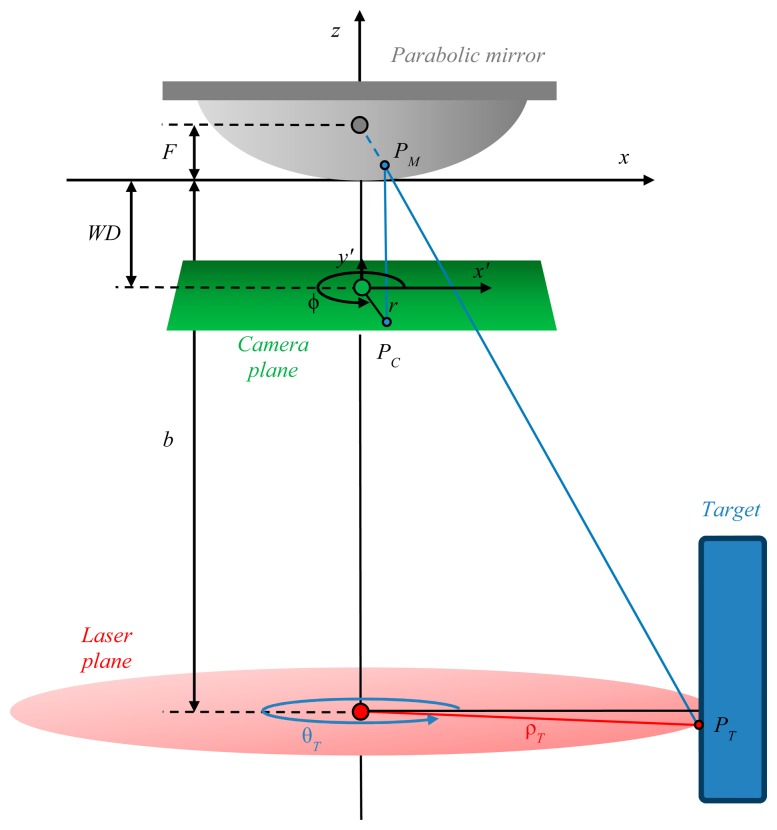
Schematic view of the proposed setup. The final goal of the analytical model is the translation of the known coordinates (*r_C_, ϕ_C_*), recovered on the camera plane, in world coordinates (ρ*_T_*, θ*_T_, z_T_*), having origin in the mirror vertex.

The aim of the proposed range sensor is the measurement of distances starting from the inspection of where the laser line is displayed in the image. The next steps are derived following the notation reported in Figure 2, where the setup scheme is proposed. Here the reference system (*x*, *y*, *z*) is centered in the vertex of the parabolic mirror, having symmetry axis along the z-direction and focus at coordinates (0, 0, *F*). It follows that the parabolic mirror has equation:

(1) $$z=\frac{1}{4F}\left(x^{2}+y^{2}\right)$$

The laser plane intercepts the *z*-axis at *b* (baseline), whereas the camera plane intercepts the *z*-axis in *WD* (working distance). For the sake of simplicity, Cartesian (*x*, *y*, *z*) and polar (ρ, θ, *z*) coordinates are both used within the next lines to refer the points in the world absolute system. Finally, the camera plane has a proper 2D reference system (*x*′, *y*′), assisted by the corresponding polar coordinates (*r*, *ϕ*).

When the laser plane strikes a target, a line emerges. Each point *P_T_* belonging to the line has coordinates (ρ*_T_*, θ*_T_, z_T_*) and is detected on the camera plane in *P_C_*, having coordinates (*r_C_, ϕ_C_*) in the reference system of the camera (*x*′, *y*′). In summary, the problem can be formulated in deriving the world coordinates (ρ*_T_*, θ*_T_, z_T_*) knowing the terms (*r_C_, ϕ_C_*). The following steps start from two fundamental initial hypotheses:

1. Both the laser and camera planes suffer from negligible alterations of their normal vectors with respect to the *z*-axis. This implies that the laser line is always across from the focus of the paraboloid, behind the camera. Equivalently each point of the line can be detected on the camera plane whenever its sight is not occluded by other objects;
2. The *z*-axis crosses the image plane in its center, or equivalently the vertex of the mirror is displayed in the center of the image plane. This condition will lead to a simplification of the model, as the image projection can be referred in both absolute and camera reference systems. In other words, the point *P_M_* is projected in *P_C_* keeping the transversal coordinates ${\left(x_{C},y_{C}\right)|}_{\left(x,y,z\right)}={\left(x_{C}^{'},y_{C}^{'}\right)|}_{\left(x',y'\right)}$. The last condition is valid when the magnification *M* of the lens does not scale the metric coordinates. Otherwise, the term *M* has to be added to the formulation as a multiplicative factor.

It is easy to understand that the calibration phase has to be run to ensure the meeting of the initial hypotheses. As an example, the mirror has to be placed properly in order to achieve its centering in the image plane. These procedures will be further described in Section 3.

Any generic point *P_T_* of the laser line produces a reflection on the parabolic mirror at coordinates defined by the point *P_M_*. Because of the properties of a parabolic mirror, the projection of the laser spot onto the mirror is equal to the intersection of the ray that connects the spot itself with the focus of the paraboloid. This ray has equations:

(2) $$\{\begin{array}{ll} x & =\frac{\rho_{T}\cos\theta_{T}\left(F-z\right)}{F+b}\\ y & =\frac{\rho_{T}\sin\theta_{T}\left(F-z\right)}{F+b} \end{array}$$

The corresponding analytical system, result of the ray incidence on the mirror, admits two solutions of *P_M_*:

(3) $$P_{M,1}=\left(\begin{array}{l} -\frac{2F\cos\theta_{T}}{\rho_{T}}\left(F+b+\sqrt{\rho_{T}^{2}+{\left(F+b\right)}^{2}}\right)\\ -\frac{2F\sin\theta_{T}}{\rho_{T}}\left(F+b+\sqrt{\rho_{T}^{2}+{\left(F+b\right)}^{2}}\right)\\ \frac{F}{\rho_{T}^{2}}{\left(F+b+\sqrt{\rho_{T}^{2}+{\left(F+b\right)}^{2}}\right)}^{2} \end{array}\right),P_{M,2}=\left(\begin{array}{l} -\frac{2F\cos\theta_{T}}{\rho_{T}}\left(F+b-\sqrt{\rho_{T}^{2}+{\left(F+b\right)}^{2}}\right)\\ -\frac{2F\sin\theta }{\rho_{T}}\left(F+b-\sqrt{\rho_{T}^{2}+{\left(F+b\right)}^{2}}\right)\\ \frac{F}{\rho_{T}^{2}}{\left(F+b-\sqrt{\rho_{T}^{2}+{\left(F+b\right)}^{2}}\right)}^{2} \end{array}\right)$$

Both solutions are valid in the set of real numbers, but only one of them is physically possible. In particular, the geometry of the system imposes a strict constrain: only that point that hits the mirror at the lowest *z*-coordinate is solution of the analytical system. It follows that *P_M_*_,2_ (from now on *P_M_*) solves the specific problem. Consequently, the coordinates of *P_C_* on the camera plane are:

(4) $$P_{C}=\left(\begin{array}{l} M\frac{2F\cos\theta_{T}}{\rho_{T}}\left(\sqrt{\rho_{T}^{2}+{\left(F+b\right)}^{2}}-\left(F+b\right)\right)\\ M\frac{2F\sin\theta }{\rho_{T}}\left(\sqrt{\rho_{T}^{2}+{\left(F+b\right)}^{2}}-\left(F+b\right)\right)\\ -WD \end{array}\right)$$

being *WD* the nominal working distance of the lens-camera set. The transversal coordinates can be also expressed in polar coordinates, thus giving the couple (*r_C_*, *ϕ_C_*) equal to:

(5) $$\{\begin{array}{ll} r_{C} & =M\frac{2F}{\rho_{T}}\left(\sqrt{\rho_{T}^{2}+{\left(F+b\right)}^{2}}-\left(F+b\right)\right)\\ {\text{f}}_{C} & =\theta_{T} \end{array}$$

Since the final goal of the presented framework is the estimation of (ρ*_T_*, θ*_T_, z_T_*) knowing the terms (*r_C_, ϕ_C_*), the relationships in Equation (5) have to be inverted, thus obtaining:

(6) $$\{\begin{array}{ll} \rho_{T} & =\frac{4MF\left(F+b\right)}{4M^{2}F^{2}-r^{2}}r=\frac{1+4ab}{1-4a^{2}r_{C}^{2}}r_{C}\\ \theta_{T} & =\phi_{C} \end{array}$$

where *a* is the curvature of the parabolic mirror, equal to $\frac{1}{4F}$.

The results in Equation (6) are thus able to transfer the points belonging to the laser line detected on the camera plane in an absolute reference system.

### 2.3. Design Strategy

Starting from the deep knowledge of the triangulation laws in Equation (6), a prototype can be designed in terms of selection of active devices, namely camera and laser sources, and passive components, *i.e.*, telecentric lens and parabolic mirror. The geometrical and physical parameters involved in the actual design of the experimental setup are reported in Table 2.

The choice of the model parameters in Table 2 is linked to a set of initial specifications:

- the maximum measurable range *d_MAX_*;
- the maximum acceptable uncertainty in range estimation Δρ*_T,MAX_* obtained at ρ*_T_* = *d_MAX_*;
- the number of profiles per second that are returned by the sensor (herein Profile Acquisition Rate, *PAR*).

The estimation of the device parameters starts with the analysis of the specified *PAR*. In particular, this requirement defines a first and unavoidable constrain on the choice of the camera, which is the only one device responsible for the measurement rate. On the other hand, the requirements on the measurement quality have effects on the choice of the mirror equation, in terms of its curvature *a*, and of the baseline *b* between mirror and lasers. Also the lens magnification *M* has to be defined properly in order to adapt the properties of the camera (pixel size and resolution) to the specific problem under analysis.

**Figure 3.**
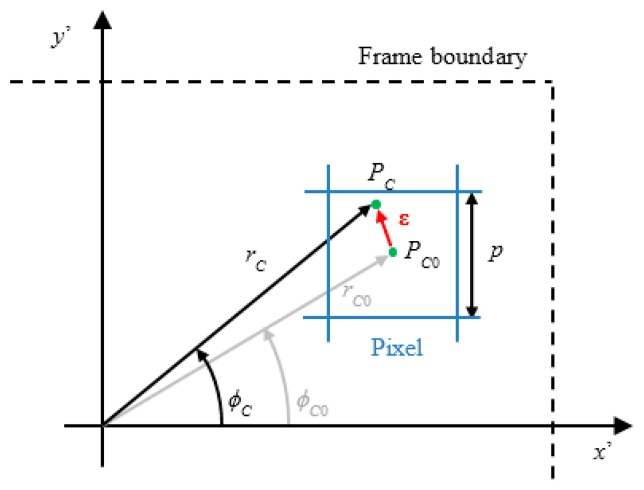
Error components related to the quantization of the image plane due to the finite area of pixels. Each pixel of the plane is square and has side equal to the pixel pitch *p*.

In this context, errors are ascribable to the quantization induced by the matrix of pixels on the camera plane. Figure 3 shows a sketch of the quantization and the corresponding effects on the determination of the beam coordinates. In particular, for any point *P_C_*, projection of the laser line within the pixel area, the resulting actual coordinates (*r_C_*, *ϕ_C_*) are always confused with the coordinates of the center of the illuminated pixel (*r_C_*_0_, *ϕ_C_*_0_). The error contribution can be described by the vector **ε**, which has origin in the center of the pixel *P_C_*_0_ and ends in *P_C_*, corresponding to the actual range measurement.

The pixel area determines a region of uncertainty. This region can be shifted in the absolute reference system, thus defining an ambiguous spatial region where differences in (ρ*_T_*, θ*_T_*) cannot be resolved. In this case, the measurement is:

(7) $$\{\begin{array}{ll} \rho_{T} & =\rho_{T0}+\Delta \rho_{T}\\ \theta_{T} & =\theta_{T0}+\Delta \theta_{T} \end{array}$$

where Δρ*_T_* and Δθ*_T_* refer to the range and angular uncertainties.

The following formulations aim to detect the worst condition for the measurement, or equivalently the highest contribution of the error vector **ε** to the couple of coordinates (*r_C_*, *ϕ_C_*). It is easy to understand that the vector **ε** has maximum modulus when the point *P_C_* exactly lies on the corners of the pixel area. In this case the modulus is equal to half the diagonal of a pixel, *i.e.*,:

(8) $${\left|ɛ\right||}_{\begin{array}{l} \Delta \rho_{T}=\Delta \rho_{T,\text{MAX}}\\ \Delta \theta_{T}=\Delta \theta_{T,\text{MAX}} \end{array}}=\frac{p\sqrt{2}}{2M}$$

It is mandatory to observe that the pitch term *p* in Equation (8) has been divided by *M* before being reported in the world reference system. In the following lines, the ratio *p*/*M* will be named as effective pixel size *p*′.

In a similar manner, when *P_C_* lies on the corners of the pixel area, the uncertainty in the determination of θ*_T_* experiences its lowest or highest values. Also in this case, the peak of uncertainty is reached along the pixel diagonal, which represents the maximum range of angles that can be spanned within the pixel itself.

In summary, given the extension of the pixel diagonal and the analytical model derived before, the maximum error can be directly estimated at a specific region of the mirror, or, equivalently, at each distance from the laser sources.

Following Equation (6), the generic pixel of coordinates (*r_C_*_0_, *ϕ_C_*_0_) corresponds to a target placed at position:

(9) $$\{\begin{array}{ll} \rho_{T0} & =\frac{1+4ab}{1-4a^{2}r_{C0}^{2}}r_{C0}\\ \theta_{T0} & =\phi_{C0} \end{array}$$

As effect of the image quantization, the returned measurement is affected by the two contribution of uncertainty, Δρ*_T_* and Δθ*_T_*. Given the hypothesis in Equation (8), the expression of Δρ*_T_* can be easily derived as:

(10) $$\Delta \rho_{T}=\frac{1+4ab}{2}\frac{2r_{C0}+p'\sqrt{2}}{1-a^{2}{\left(2r_{C0}+p'\sqrt{2}\right)}^{2}}-\rho_{T0}$$

which leads to:

(11) $$\Delta \rho_{T}=\frac{p'\sqrt{2}}{2}\frac{1+4ab}{1-4a^{2}r_{C0}^{2}}\frac{1+2a^{2}\left(2r_{C0}+p'\sqrt{2}\right)r_{C0}}{1-a^{2}{\left(2r_{C0}+p'\sqrt{2}\right)}^{2}}$$

Equation (11) can be further manipulated to derive the expression of Δρ*_T_* as a function of the measurement ρ*_T_*_0_. This result can be easily obtained inverting the first equation of Equation (9):

(12) $$r_{C0}=\frac{\sqrt{{\left(1+4ab\right)}^{2}+16a^{2}\rho_{T0}^{2}}-\left(1+4ab\right)}{8a^{2}\rho_{T0}}$$

At the same time, the maximum angular uncertainty in target measurements can be derived knowing the coordinates of the point *P_C_* and *P_C_*_0_ in the *x*′*y*′-plane and how they are related to the pixel size. For instance, if *P_C_* lies on the north-west corner of the pixel depicted in Figure 3, it is possible to derive Δθ*_T_* as follows:

(13) $$\Delta \theta_{T}=\arctan\left(\frac{2r_{C0}\sin\theta_{T0}+p}{2r_{C0}\cos\theta_{T0}-p}\right)-\theta_{T0}$$

which can be further developed as a function of the range measurement, by replacing the expression in Equation (12).

Equations (11)–(13) are necessary but not sufficient to achieve the complete design of the sensor, which requires the last constrain: the mirror has to be in the FoV of the selected camera. When the mirror is acquired by the camera, its edges define a circle of diameter *D_M_*. It is easy to understand that this area has to be included within the camera plane in order to be captured, and, consequently, the mirror diameter has to be at least equal to the smallest size of the camera sensor. Specifically, being *W* and *H* the number of pixels along the horizontal and vertical directions (*H* ≤ *W*), *D_M_* has to be equal to *h* = *H*·*p*′.

Since the sensor has to return measurements at a maximum distance *d_MAX_* from the laser sources, Equation (12) can be rewritten imposing that a laser beam, impinging on a target at distance *d_MAX_*, is detected on the most external regions of the mirror. Mathematically, this condition leads to impose that $r_{C0}=\frac{D_{M}}{2}$ when ρ*_T_*_0_ = *d_MAX_*. This can be exploited to define the unknown baseline *b* as a function of the mirror curvature *a*:

(14) $$b=\frac{2\left(1-a^{2}h^{2}\right)d_{\text{MAX}}-h}{4ah}$$

As a consequence, the design can be shifted to the evaluation of the unknown *a*, which is the only term that has to be dimensioned to match the specification on the maximum error. Equation (11) can be developed considering ${r_{C0}|}_{\rho_{T0}=d_{\text{MAX}}}=\frac{D_{M}}{2}=\frac{h}{2}$, together with the expressions Equations (8) and (12), thus obtaining:

(15) $$a=\sqrt{\frac{h\Delta \rho_{T,\text{MAX}}-p'\sqrt{2}d_{\text{MAX}}}{h\left(h+p'\sqrt{2}\right)\left(h\Delta \rho_{T,\text{MAX}}+p'\sqrt{2}\left(d_{\text{MAX}}+\Delta \rho_{T,\text{MAX}}\right)\right)}}$$

Note that only the positive solution of *a* has been considered, accordingly with the sketch in Figure 2, where a concave up paraboloid is presented.

In summary, the first specifications on the maximum measurement range and the maximum acceptable error define univocally the geometrical parameters that determine the shape of the parabolic mirror, in terms of its curvature *a* in Equation (15), and the position of the laser sources along *z*, assessed by the baseline *b* in Equation (14).

## 3. Experimental Analysis

### 3.1. Prototype Description

As described in details, the proposed range sensor is based on the principle of laser triangulation. Following the early idea given in [46–48], the triangulation process is assisted by a parabolic mirror in order to achieve a wide FoV of 270°.

The aim of this investigation is a further improvement of the previous setup in terms of the reduction of the sensor size and the increase of the measurement rate. Specifically, the first prototype implements a parabolic mirror whose radius is equal to 60 mm. The corresponding telecentric lens, chosen to capture the whole mirror area, has the same radius of the reflector, and a length of about 600 mm. At the same time the distance between the vertex of the mirror and the laser plane, from now on baseline *b*, has been dimensioned equal to 1.5 m to acquire measurements with a maximum relative error of 0.1% at a distance of 3 m from the emitters. Finally the *PAR*, which determines the number of slices per seconds that maps the environment, is equal to 5.

The novel design fixes new initial specifications. As a first step the setup has to be reduced in size to a maximum total length of 1 m, keeping the measurement resolution Δρ*_T,MAX_* to 10 mm at a maximum distance *d_MAX_* of 3 m. At the same time the *PAR* has to be improved reaching 25 profiles per second. These aspects imply the use of state-of-art devices, together with the redefinition of the design parameters, to fit the new requirements.

**Figure 4.**
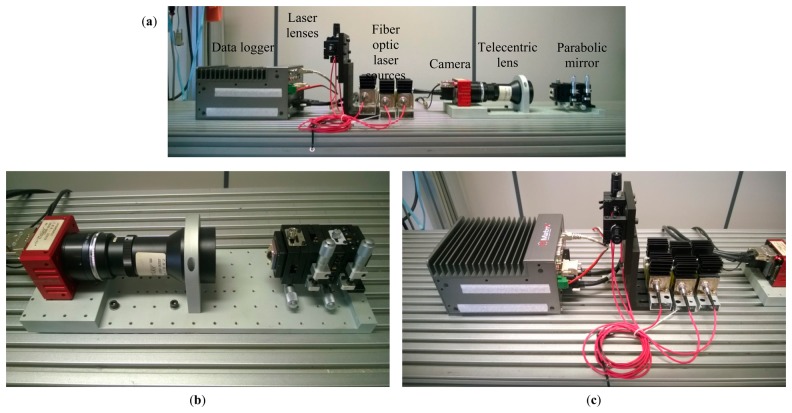
Picture of the actual prototype: (**a**) Overall setup; (**b**) Optical receiver made of the parabolic mirror and the lens-camera set; (**c**) Laser sources and lenses and data logger connected to the camera.

With reference to Figure 4, where a first prototype is presented, the sensor exploits fiber optic lasers, namely CUBE Laser by Coherent [49], with a built-in thermal management. Furthermore, the use of fiber tails assisted by cylindrical lenses enables the reduction of the space required for its mechanical assembling. At the same time, the initial specification of high measurement rate is ensured by the use of the CL-400 Bonito camera by Allied Vision Technology [50], which exploits the double and full Cameralink protocol with frame rates *f* up to 386 frames per second. The main features camera are reported in Table 3.

**Figure 5.**
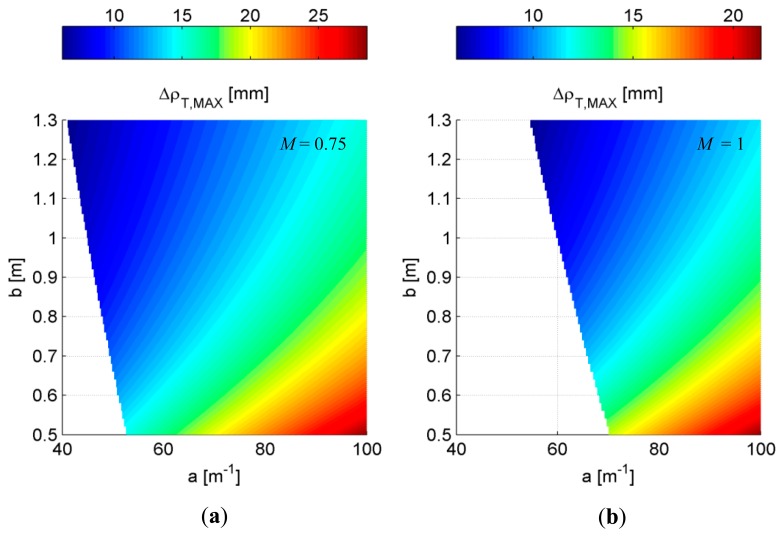
Maximum errors Δρ*_T,MAX_* at *d_MAX_* = 3 m as a function of the mirror curvature and the baseline, computed for magnification equal to: (**a**) 0.75; (**b**) 1. Regions where the laser incidence is out of the camera FoV are displayed in white.

Once the camera has been selected, the unknowns *a*, *b* and *M* have to be dimensioned to match the initial specifications on the measurement error and sensor size. As stated previously, the error analysis leads to Equations (14) and (15) which can be easily exploited to derive the mirror curvature and the baseline, as a function of the magnification of the telecentric lens, implicitly held in *p*′. Typical values of the magnification *M* are 0.75, 1 and 2 (e.g., see [51]). These numbers have been tested, producing the results in Figure 5, where the maximum error Δρ*_T,MAX_* is reported as a function of the mirror curvature and the laser-mirror distance. The presented plots are computed for realistic values of *a* and *b*. Specifically, the mirror curvature spans describing a maximum mirror depth of about 13 mm, corresponding to *a* = 100 m^−1^ with *M* = 0.75. It is important to notice that high-curvature mirrors are not suitable for the specific application, since their depths are much over the limit imposed by the depth of field of the lens, typically close to few millimeters. In this case, the telecentric lens is not able to focus the mirror over its entire depth, or equivalently over its whole area. At the same time, the trial values of the baseline are limited to 1.3 m, anyway higher than the desired maximum length of the sensor.

Before going through the inspection of Figure 5, it is worth observing that, within the considered boundaries of *a* and *b*, a magnification *M* equal to 2 does not produce visible reflections on the mirror, *i.e.*, in the camera FoV, when the target is 3 m far from the laser sources. This value defines the maximum range of the proposed device, which makes it most suitable for indoor applications. At the same time, those outdoor applications where the main interest is focused on the closest targets (see railway monitoring) can be faced, taking advantage of the coherent nature of the laser line, which is highly recognizable against the ambient light. Nevertheless, also higher maximum ranges can be reached by changing properly the optical components involved in the presented setup.

The insight of Figure 5 demonstrates that when the magnification is equal to 0.75, the lower values of *a* and *b* that allows Δρ*_T,MAX_* = 10 mm are 48.22 m^−1^ and 756.9 mm, respectively. On the other, the same specification is matched for *a* = 64.29 m^−1^ and *b* = 758.2 mm, when *M* = 1. Although baselines are almost equal, the mirror curvatures change considerably. As stated previously, a conscious design would prefer lower curvatures, since the corresponding mirrors have shorter depths. In this way, the telecentric lens can extend its working distance over increasing areas of mirror, keeping the laser line focused. Hence, the telecentric lens VS-LTC075-70-35/FS by VS-Technology [51], having magnification equal to 0.75, has been chosen for the presented prototype.

The final design parameters that allow the specification compliance are thus summarized in Table 4.

**Figure 6.**
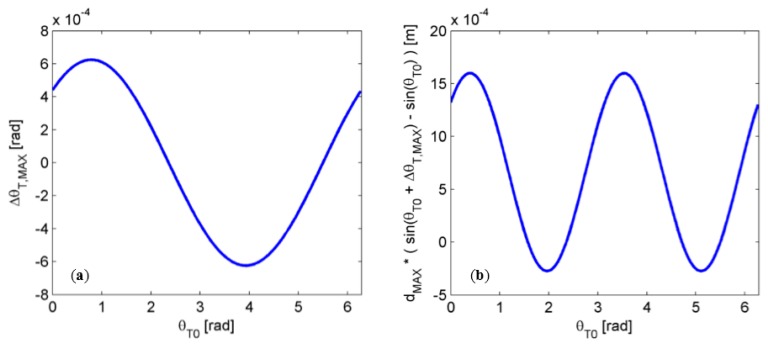
(**a**) Angular component of the maximum measurement error at *d_MAX_* = 3 m as a function of the estimated angle θ*_T_*_0_; (**b**) Maximum estimated shift, due to the presence of angular uncertainty Δθ*_T,MAX_*, in the computation of the *y*-coordinate of the point *P_C_* at *d_MAX_* equal to 3 m.

Once the design parameters have been selected, the maximum error in the computation of the angular component of *P_T_* can be estimated. With reference to Equation (11), this error contribution depends on the exact angular component of the point *P_C_*_0_. Figure 6(a) shows the dynamics of the error term Δθ*_T,MAX_* as a function of the angle θ*_T_*_0_, equal to *ϕ_C_*_0_.

The analysis of Figure 6 demonstrates that the angular component of the maximum error due to image quantization is always below 3.5 × 10^−2^ degrees. As a consequence, the estimation of the target position in the (*x*, *y*, *z*) system of coordinates is altered as results of the application of sine and cosine functions to the term θ*_T_*_0_ + Δθ*_T,MAX_*. Quantitatively the maximum error due to Δθ*_T,MAX_* in determining the *x* and *y* coordinates of the point *P_C_* is at most equal to l.6 mm at a distance of 3 m (see Figure 6b), *i.e.*, about one order of magnitude lower than the specified Δρ*_T,MAX_*.

### 3.2. Setup Calibration

However precise and mechanically stable the experimental setup can be, the actual geometrical parameters differ from the nominal ones. As a consequence, the setup calibration has to compensate for this, estimating the unknown parameters *F* (or equivalently *a*) and *b* that govern the triangulation process. This task is mandatory within a calibration phase, which is driven by the inspection of a completely-known target.

Before going through the estimation, it is important to mention the preliminary assumption of the model, regarding the relative position of the mirror and the image plane. Specifically, the camera plane has to intercept the axis of symmetry of the mirror in its center. Since the camera has greater sizes than the mirror, it is more convenient to change the position of the latter, keeping the camera fixed at a distance from the mirror close to *WD*. Consequently, the mirror has been bracketed on mechanical handles, taking advantages of micrometric shifts in the *xy*-plane and rotations around the *x*- and *y*-axis.

**Figure 7.**
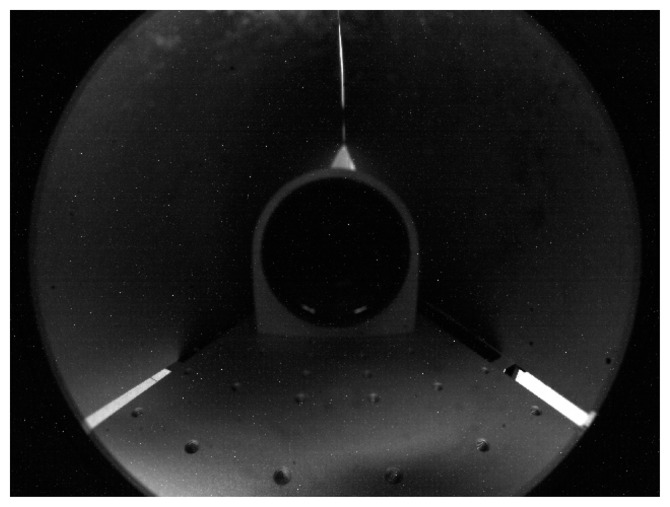
Example of frame captured by the camera for the estimation of the mirror position in the image plane.

Once the mirror has been equipped with micrometric rototranslational stages, a processing pipeline is needed to estimate its position within the image plane. The algorithm of mirror identification has been developed in the MVTech Halcon 11 [52] environment. In this case, the position of the mirror vertex can be estimated by searching for the mirror circular boundary in a set of sample frames captured by the camera. Figure 7 shows an example of image returned by the camera, where a self-reflection of the telecentric lens can be observed in the image center, whereas the mirror boundary can be easily recognized on the outer regions.

**Figure 8.**
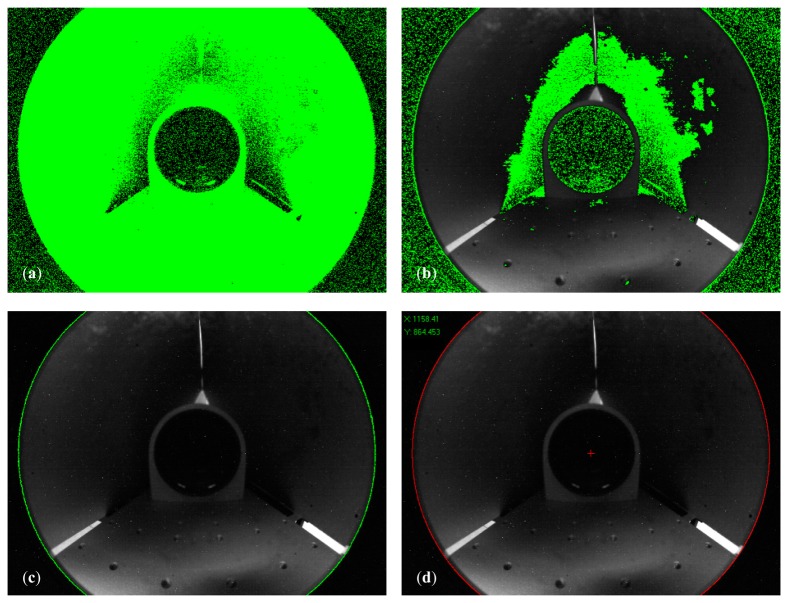
Image processing steps for the determination of the mirror position in the camera plane. (**a**) Threshold image; (**b**) Boundaries extracted from the threshold regions of high intensity; (**c**) Start image and corresponding fitting ellipse (in green); (**d**) Final results with the estimation of the center coordinates (red cross).

With reference to Figure 8, where the contour extraction is presented step by step, the implemented algorithm processes the returned frames (e.g., Figure 7) to estimate the mirror position through the following steps:

(1) A process of image threshold highlights the pixels of intensity higher than 20, returning the green area in Figure 8a;
(2) Given the areas of high intensity, the method extracts the region contours in Figure 8b;
(3) The longest boundary is selected and fitted on a two-dimensional ellipse in the least squares sense, producing the green curve in Figure 8c;
(4) The center coordinates are consequently derived (red cross in Figure 8d), whereas the eccentricity of the estimated ellipse is evaluated to measure the alteration of the normal vector of the image plane with respect to the *z*-axis.

The presented algorithm controls the mirror position in real time, thus enabling the direct use of the micrometric stages for its exact placement. In this way, the initial hypothesis that leads to the model in Equation (6) is verified.

**Figure 9.**
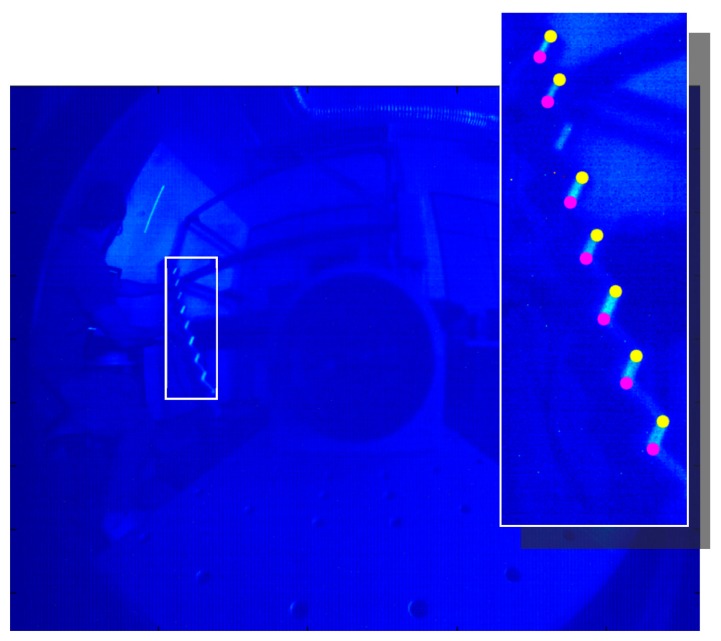
Example of a frame captured by the camera during the setup calibration. The laser line illuminates a target of completely known geometry. The inset highlights the extracted points belonging to the structure edges, whose distance corresponds to the thickness of the wood strips.

The calibration phase can thus proceed with the estimation of the unknown model parameters. For this purpose, a wood structure made of 45-mm-thick strips has been realized and scanned by the proposed sensor in order to frame couples of laser points belonging to the strip corners. The Euclidean distance computed in the image plane between corresponding corner points is then compared to the actual corner distance, implicitly equal to the thickness of the laths. Figure 9 reports an example of an acquired frame used for the setup calibration, whereas the inset shows the corresponding couple of points named as the structure edges.

The experimental calibration is treated as an optimization problem. As a first step the couples of edges are extracted, passing through the following steps:

(1) The image is cropped in order to eliminate secondary reflections due to the presence of external light sources, returning the region enclosing the sample target (see the inset of Figure 9);
(2) The ROI is treated by a threshold process to highlight the laser points. This step generates a binary image where white pixels are candidate laser points;
(3) A region growing approach is applied, after a morphological dilation filter, to detect continuous region that resamples the laser line;
(4) The resulting regions are individually fitted on an ellipse. The limits of the major axis determine the edges of the laser line impinging on the sample target. These points are derived with subpixel resolution.

Once the edges are extracted, these are transformed in world coordinates, using trial parameters. An objective cost function is thus defined as the square error between the computed edge distances and their nominal counterparts. The problem is thus solved in the non-linear least squares sense.

An overdetermined system is built exploiting more than 100 frames and solved in the model parameters, thus obtaining the resulting values in Table 5, with a corresponding residual of the cost function of 3.205 × 10^−4^.

The actual values of the model parameters determine a drift of the measurement obtained under ideal conditions. From a quantitative point of view, compensating for the presence of setup alterations allows the deletion of additional systematic errors. With more details, considering the nominal parameters instead of the calibrated ones generates a peak error of 144.19 mm at the maximum range of 3 m, *i.e.*, one order of magnitude higher than the required range resolution (10 mm).

### 3.3. Experiments and Discussions

The experimental validation of the sensor setup can be performed in two different ways:

(1) Inspecting the movement of a target, which is mechanically controlled via encoded slits and rotational stages. This technique requires the perfect understanding of the mathematical relationships between the world reference system, where the target shift is defined, and the mirror reference system, where actual results are determined;
(2) Scanning the shape of a known object, placed at increasing distances from the laser sources. This method returns relative measurements, which are characteristics of the target itself. It follows that the knowledge of the object pose in the mirror system of coordinates is no longer required. The comparison is self-consistent, given the shape of the target.

For these reasons, several acquisitions have been performed with the aim of determining the size of a square board, placed at increasing distances. Moreover, experiments have been run changing the direction of the radial shifts in order to cover many spatial regions. This results will be of interest since the goal of the proposed system is the inspection of surroundings, wrapped around the range sensor. In this case, it is mandatory to ensure that the measurements are always reliable, regardless the target position.

**Figure 10.**
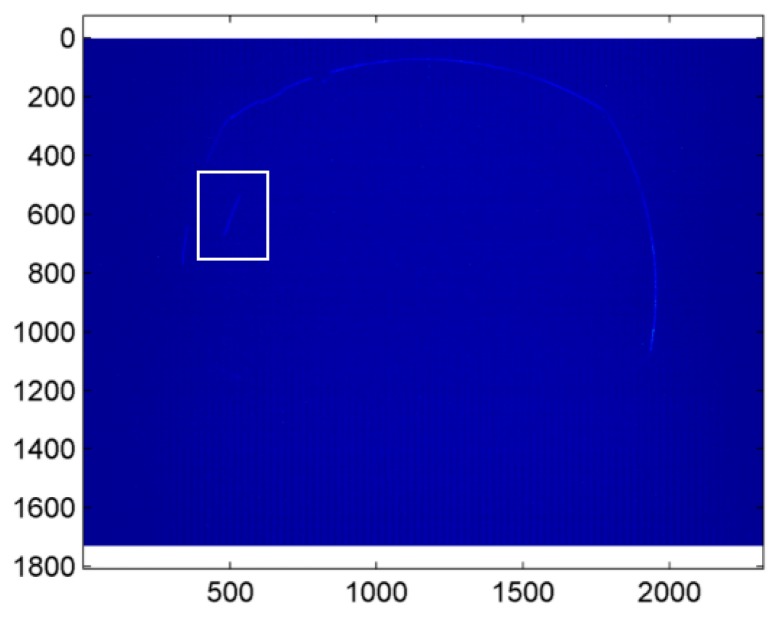
Example of frame acquired by the camera for testing the sensor accuracy. The rectangle encloses the laser line impinging on the board.

**Figure 11.**
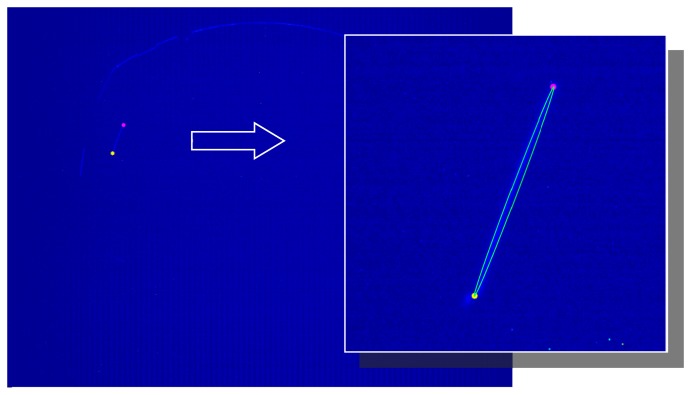
Results of the edge extraction algorithm used for the detection of the board sizes. The inset shows a magnified view of the extracted points; the green line identifies the fitting ellipse.

Figure 10 reports an example of frame, acquired when the laser line impinges on a square paperboard having side equal to 310 mm. The base of the board has been perfectly aligned to the ground, in order to ensure that the line crosses it parallel to its vertical sides. The edges of the laser line have been extracted by means of the same algorithm used in the calibration phase for the corner extraction from the known target. In summary, a ROI including the laser line is extracted and a binary image is built by means of a threshold process; after the application of a dilation filter, a region growing approach is used to determine the actual laser line, which is fitted on an ellipse. The edges of the laser line on the board sample are equal to the limits of the major axis of the fitting ellipse. The result of the proposed algorithm, applied to the frame of Figure 10, are shown in Figure 11.

**Figure 12.**
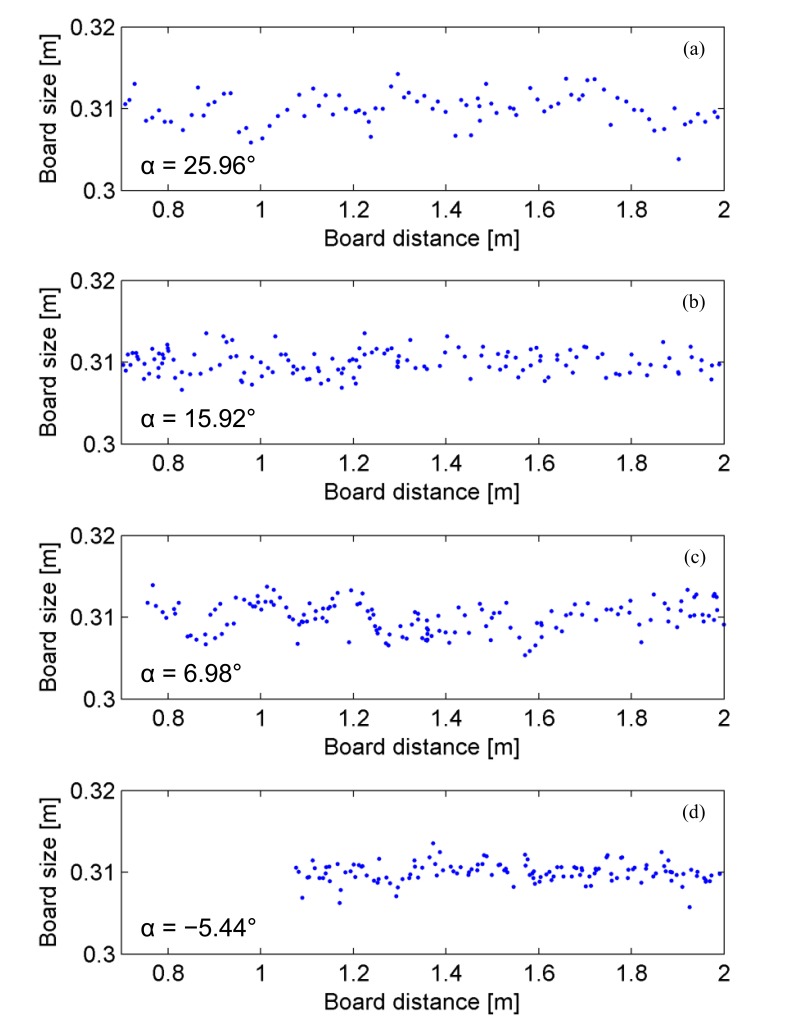
Estimation of the size of the sample board as a function of the distance of the target from the light sources. Measurements have been performed changing the direction of the radial shifts, accordingly with the axis defined by the angle α, referred to the ground plane: (**a**) α = 25.96°; (**b**) α = 15.92°; (**c**) α = 6.98°; (**d**) α = −5.44°.

Once the edges of the laser line are extracted from the image, they can be reported in the (*x*, *y*, *z*) reference system, thus obtaining their positions in space. It is evident that the spatial distance between the edges is implicitly equal to the side of the panel. Figure 12 points out the estimated dimension of the board as a function of the target distance. Plots are obtained spanning the target movement around the sensor for discrete angles *α*, which defines the direction of the target shifts with reference to the ground (assumed parallel to *xz*-plane).

Results clearly show the good agreement of results in computing the dimension of the board side, regardless the target position, which qualitatively does not alter the measurement error. In particular the average values of the estimated dimensions of the board are reported in Table 6.

**Figure 13.**
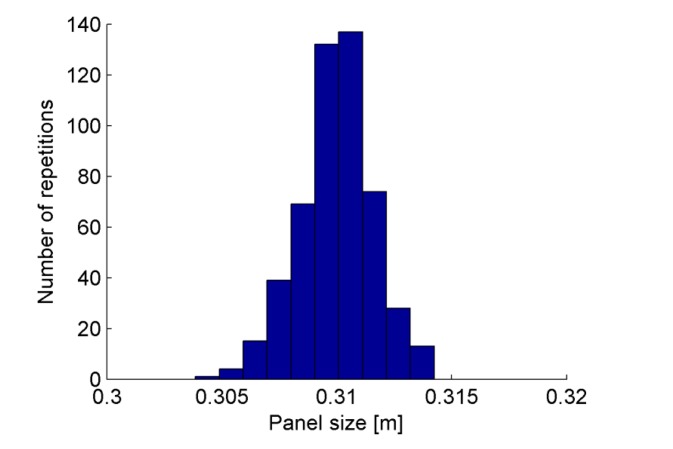
Collection of samples returned by the analysis of the board side.

Moreover, range samples have been collected in equally spaced bins in order to derive information about the noise statistics, leading to Figure 13. At this stage, quantization errors are compensated by the process of point extraction, which computes the position of the panel borders with subpixel precision. Here, the main contributions to the measurement errors are related to a superposition of two mechanisms of degradation. First the laser line is defocused on the camera plane as effect of the finite depth of field of the camera and the divergence of the laser light. Then, the image processing introduces implicit approximations, since curve lines corresponding to straight segments are actually fitted by ellipses. Nevertheless, the data collection in Figure 13 follows a Gaussian-shaped function centered on the expected measurement, thus proving the good accuracy of the proposed sensor. Measurements are altered by noise contribution with standard deviation of 1.74 mm and a consequent ∼99% confidence interval of about 10.44 mm.

Furthermore, the presented error estimation is uncorrelated with respect to the camera frame rate, till the limit fixed by the inverse of the exposure time used in the presented experiments (30 ms). When the frame rate is higher than 33 fps, the exposure time has to be reduced properly, thus downing the intensity amplitude of the detected laser line. As a consequence, the decreasing signal-to-noise ratio can produce effects on the measurement quality. Nevertheless, the initial requirement of fast acquisitions (25 fps) can be matched within the limit of precision discussed before.

The presented results can be compared with those returned by the AccuRange AR4000 rangefinder, whose range measurements are affected by a statistical white noise with standard deviation of 2.5 mm when the target is placed 1 m far from the emitter [53]. Although noise contributions seem comparable, the frame rate of the AR4000 rangefinder imposed for these experiments is equal to 1 kHz. On the other hand, the presented sensor produces about 5 × 10^4^ samples per second at the current frame rate of 25 fps. This behavior is due to the camera resolution which allows the proper decomposition of the detected laser line of a single frame in more than 2000 samples, without any degradation of the measurements.

### 3.4. 3D Reconstruction

**Figure 14.**
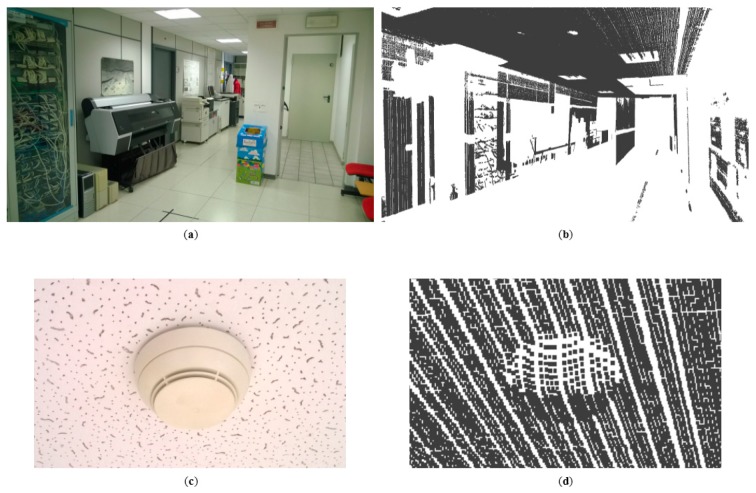
(**a**) Acquired corridor and (**b**) corresponding 3D reconstruction; (**c**) picture of a particular object with maximum size of 10 cm and (**d**) corresponding 3D model.

As a proof of the actual capabilities of the presented range sensor in 3D reconstruction, an example of acquisition is briefly reported in this Section. The sensor is fastened on a mobile robot, which flows through an indoor environment (in this example a corridor) following straight trajectories at a constant speed of 400 mm/s. The camera is trigged by a TTL signal generated by the robot encoders. Given the resolution of the encoders and the robot speed, the camera sends a frame to the data receiver every 5 mm, exploiting the full camera link protocol. This data is a raw matrix with 1728 × 2320, full of unsigned char representing the image intensities. Frames are then processed to extract the position of the laser line in the image plane. At this stage, the image is sectioned following 2048 radial directions, starting from the image center. Each section can include at most one laser peak, whose position can be easily computed applying the standard center of mass approach [54]. Knowing the exact position of the laser line with subpixel accuracy and the robot pose returned by odometry, it is possible to derive the corresponding coordinates in three-dimensions. These samples are finally ordered in a Wavefront .obj file, filled by the vertex of the dataset. The reconstruction has produced a point cloud having size equal to 2.4 × 10^6^. This outcome is shown in Figure 14.

## 4. Conclusions

In this paper, an omnidirectional range sensor for the inspection of surrounding spaces had been developed. Following the principles of laser profilometry, the range sensor estimates the distance of targets by looking at the displacements of a laser line projected onto the environment. When the vision system is assisted by a parabolic mirror, high FoV can be reached in a single scan, *i.e.*, a camera frame, thus increasing the number of profiles, up to the limit fixed by the camera electronics. The experimental setup had been designed following analytical expressions to meet initial specification on its overall size and the measurement resolution at a distance of 3 m from the emitters. A novel calibration phase devoted to the alignment of the optical component involved in the acquisition had been described, together with the estimation of the actual geometrical parameters that lead to the range measurements. Several experiments had been run in order to establish whether the proposed system can inspect accurately the surface of known calibrated targets, using effective image processing techniques. Measurements returned by the sensor for the estimation of the size of the known target had been compared with nominal values. Experimental results had demonstrated that the noise contribution follows a Gaussian shape with standard deviation of 1.74 mm and negligible systematic error (mean value close to 0.31 mm), regardless the target distance from the sensor. All noise sources are ascribable to the defocusing effect induced by the finite depths of field of both emitting lasers and receiving system. Keeping the same exposure of the camera, the profile acquisition rate can reach 33 profiles per second, as required by the specifications, without increasing the maximum error. In conclusion, the presented device constitutes one of the best options for applications, such as inspection of pipes or monitoring of confined spaces, where reliable range measurements with high acquisition rates, high resolution and accuracy are mandatory.

## Figures and Tables

**Table 1.** List of available devices for 3D reconstruction of environments.

**Model Name**	**Accurange AR4000 [22]**	**RIEGL VQ-250 [23]**	**RIEGL VZ-400 [23]**	**Optech ILRIS [24]**	**Bumblebee BB2-08S2 [25]**	**Kinect v2 [26]**
*Type*	Laser Scanner	Laser Scanner	Terrestrial Scanner	Terrestrial Scanner	Depth camera	Depth camera
*Acquisition rate*	50 kHz	50 kHz	122 kHz	10 kHz	1032 × 776 @ 20 Hz	512 × 424 @ 30 Hz
*Maximum distance*	2 m	180 m	350 m	400 m	10 m	4.5 m
*Resolution*	5 mm	Not reported	Not reported	Not reported	Not reported	2 mm
*Accuracy*	5 mm @ 9 m	10 mm	5 mm	4 to 7 mm @ 100 m	Not reported	1 mm
*Precision*	Not reported	5 mm	3 mm	Not reported	Not reported	Not reported
*(I)n/(O)utdoor*	I/O	I/O	O	O	I/O	I
*Applications*	3D Environment reconstruction	Mobile mapping from moving platforms	Large environments reconstruction end inspection	Large environments reconstruction end inspection	3D Environment modeling	3D Environment modeling

**Table 2.** List of geometrical and physical parameters involved in the range measurements.

**Components**		**Parameter Name**	**Description**
Passive	Mirror	*a*	Curvature of the mirror [m^−1^]
	Lens	*M*	Magnification of the lens

Active	Camera	*W × H*	Resolution of the camera
		*p*	Pixel size [m]
		*f*	Frame rate [s^−1^]

	Laser	*b*	Baseline [m]

**Table 3.** Specifications of the implemented camera (AVT CL-400 Bonito [50]).

**Parameter Description**	**Value**
Interface	2 × 10-tap CL Full+
Image resolution (*W* × *H*)	2320 × 1728
Sensor size	4/3″
Pixel size (*p*)	7 μm
Max frame rate at full resolution	386 fps

**Table 4.** List of design parameters that allow maximum error of 10 mm at a distance of 3 m.

**Parameter**	**Value**
*A*	48.22 m^−1^
*B*	756.9 mm
*M*	0.75

**Table 5.** Results of the calibration process.

**Parameter**	**Calibration Results**
*F*	5.166 mm
*B*	702.12 mm

**Table 6.** Average values of the measured size of the square paperboard under analysis. The target has a nominal dimension of 310 mm.

**α**	**Paperboard Size**
25.96°	309.77 mm
15.92°	310.62 mm
6.98°	310.23 mm
−5.44°	309.39 mm
